# Genetic Polymorphisms of *IL28B* and *PNPLA3* Are Predictive for HCV Related Rapid Fibrosis Progression and Identify Patients Who Require Urgent Antiviral Treatment with New Regimens

**DOI:** 10.1371/journal.pone.0137351

**Published:** 2015-09-09

**Authors:** Nobuharu Tamaki, Masayuki Kurosaki, Mayu Higuchi, Hitomi Takada, Natsuko Nakakuki, Yutaka Yasui, Shoko Suzuki, Kaoru Tsuchiya, Hiroyuki Nakanishi, Jun Itakura, Yuka Takahashi, Shintaro Ogawa, Yasuhito Tanaka, Yasuhiro Asahina, Namiki Izumi

**Affiliations:** 1 Department of Gastroenterology and Hepatology, Musashino Red Cross Hospital, Tokyo, Japan; 2 Department of Virology and Liver Unit, Nagoya City University Graduate School of Medical Sciences, Aichi, Japan; 3 Department of Hepatitis Control, Tokyo Medical and Dental University, Tokyo, Japan; Kaohsiung Medical University Hospital, Kaohsiung Medical University, TAIWAN

## Abstract

The assessment of individual risk of fibrosis progression in patients with chronic hepatitis C is an unmet clinical need. Recent genome-wide association studies have highlighted several genetic alterations as predictive risk factors of rapid fibrosis progression in chronic hepatitis C. However, most of these results require verification, and whether the combined use of these genetic predictors can assess the risk of fibrosis progression remains unclear. Therefore, genetic risk factors associated with fibrosis progression were analyzed in 176 chronic hepatitis C patients who did not achieve sustained virological response by interferon-based therapy and linked to the fibrosis progression rate (FPR). FPR was determined in all patients by paired liver biopsy performed before and after therapy (mean interval: 6.2 years). Mean FPR in patients with *IL28B* (rs8099917) TG/GG and *PNPLA3* (rs738409) CG/GG were significantly higher than in those with *IL28B* TT (FPR: 0.144 vs. 0.034, *P* < 0.001) and *PNPLA3* CC (FPR: 0.10 vs. 0.018, *P* = 0.005), respectively. *IL28B* TG/GG [hazard ratio (HR): 3.9, *P* = 0.001] and *PNPLA3* CG/GG (HR: 3.1, *P* = 0.04) remained independent predictors of rapid fibrosis progression upon multivariate analysis together with average alanine aminotransferase after interferon therapy ≥40 IU/l (HR: 4.2, *P* = 0.002). Based on these data, we developed a new clinical score predicting the risk of fibrosis progression (FPR-score). The FPR-score identified subgroups of patients with a low (FPR: 0.005), intermediate (FPR: 0.103, *P* < 0.001), and high (FPR: 0.197, *P* < 0.001) risk of fibrosis progression. In conclusion, *IL28B* and *PNPLA3* genotypes are associated with rapid fibrosis progression, and the FPR-score identifies patients who has a high risk of fibrosis progression and require urgent antiviral treatment.

## Introduction

Infection with hepatitis C virus (HCV) is a common cause of chronic hepatitis, which may eventually progress to cirrhosis and hepatocellular carcinoma[1]. Most recently, major advances in the treatment of HCV have been achieved by the development of new direct-acting antiviral agents (DAAs). However, the high cost of DAA regimens and competing public health priorities have prompted a worldwide discussion whether all patients should have access to the new therapies without restriction. In many countries, new DAA regimens are therefore reserved for patients with advanced fibrosis or cirrhosis, only. However, a scenario of waiting for the development of advanced fibrosis in patients with early stage liver disease (F0-F2) may result in an increased burden of HCV-related disease, including the development of hepatocellular carcinoma and ultimately increased cumulative costs. Thus, it is crucial to identify patients at early disease stages but high risk of fibrosis progression who would consequently require urgent HCV treatment.

This issue is of special importance for patients who did not achieve SVR in a previous course of antiviral therapy with interferon. Although the eradication of HCV by interferon alone or in combination with ribavirin improves hepatic inflammation and fibrosis[2], a substantial number of patients remain viremic even after previous interferon-based therapy because of a low sustained virological response (SVR) rate, especially in genotype 1 (<50%). However, the progression rate of fibrosis varies among these patients[3] and the assessment of individual risk of fibrosis progression in patients with chronic hepatitis C after antiviral therapy remains an unmet clinical need.

Recent genome-wide association studies (GWAS) have highlighted several genetic alterations as predictive risk factors of rapid fibrosis progression in chronic hepatitis C. A single nucleotide polymorphism (SNP) located near *interleukin 28B* (*IL28B*) that encodes type III interferon λ3 was strongly associated with interferon-induced clearance of HCV[4,5,6,7,8]. Therefore, the *IL28B* genotype may be associated with fibrosis progression after interferon-based therapy, although the results of recent studies remained inconclusive[9,10,11]. Recent European GWAS have identified a series of SNPs [*MERTK* (rs4374383), *TULP1* (rs9380516), *GLT8D2* (rs2629751), and *RNF7* (rs16851720)] as susceptible genetic alterations for HCV-related liver fibrosis[12], and other studies have proposed SNPs at rs738409 in *adiponutrin/patatin-like phospholipase domain-containing 3* (*PLPNA3*) and rs5764455 in *PARVB*, which is in strong linkage disequilibrium with *PLPNA3*, as genetic determinants of liver fat content[13], disease progression, and fibrosis in nonalcoholic fatty liver disease (NAFLD)[14,15,16,17,18,19,20]. However, most of these results require verification, and whether the combined use of these genetic predictors can assess the risk of fibrosis progression in patients with HCV after antiviral therapy remains unclear.

It was the aim of this study to identify genetic risk factors (SNPs) associated with fibrosis progression in patients with chronic hepatitis C who failed interferon-based therapy.

## Methods

### Patients

Written informed consent was obtained from each patient. The study protocol conformed to the ethical guidelines of the Declaration of Helsinki and was approved by the ethics review committees of Musashino Red Cross Hospital.

Genotyping of SNPs in *IL28B*, *PNPLA3*, *MERTK*, *TULP1*, *GLT8D2*, *RNF7*, and *PARVB* was performed in 176 patients who underwent interferon-based therapy between 1991 and 2013 at Musashino Red Cross Hospital and did not achieve SVR. All patients had undergone liver biopsies before and after interferon therapy, with a mean interval period of 6.2±3.8 years. Of the 176 patients, 64 received interferon-α or interferon-β monotherapy for 24 weeks, 61 received interferon-α/ribavirin combination therapy for 24 weeks, 8 received peginterferon-α monotherapy for 48 weeks, and 43 received peginterferon-α/ribavirin combination therapy for 48 to 72 weeks. All patients had not achieved SVR and were still HCV positive at the second biopsy. No patient had an alcohol consumption of more than 20 g per day, co-infection with hepatitis B virus or human immunodeficiency virus, or liver disease of other known etiologies such as autoimmune hepatitis or primary biliary cirrhosis.

Patients with cirrhosis at baseline were excluded because the endpoint of the study was fibrosis progression. Age was determined at the first biopsy. Laboratory tests were performed monthly or bimonthly in all patients, and all measurements were performed at a single hospital. Patients negative for HCV-RNA 24 weeks after interferon therapy completion were defined as SVR. The average value of alanine aminotransferase (ALT) after interferon therapy up to 1 year was calculated, and ALT normalization after interferon therapy was defined as average ALT of <40 IU/l.

### Histological evaluation

Laparoscopic or ultrasound-guided liver biopsy was performed using 13-gauge or 15-gauge needles, respectively. The median length of specimens was 15 mm (range: 10–30 mm), and the median number of portal tracts was 12 (range: 8–25). Specimens were fixed, paraffin-embedded, and stained with hematoxylin–eosin and Masson’s trichrome. A minimum 10-mm biopsy sample was required for diagnosis. All liver biopsy samples were independently evaluated by 2 senior pathologists who were blinded to the clinical data. Fibrosis staging was categorized according to the METAVIR score: F0, no fibrosis; F1, portal fibrosis without septa; F2, portal fibrosis with few septa; F3, numerous septa without cirrhosis; and F4, cirrhosis. When staging was inconsistent between the 2 pathologists, an appropriate stage was determined by discussion between them. The annual fibrosis progression rate (FPR) was calculated as the change in fibrosis staging divided by the time between paired biopsies. For the tentative definition of rapid fibrosis progression, a cut off value of FPR ≥ 0.2 was selected because it was upper quartile of FPR in the present study and it was approximately 1.5 times of the previously reported FPR in natural history[3]. Hepatic steatosis was graded according to the percentage of affected hepatocytes as follows, 0: absent or <5%; 1: 5%–33%; 2: 34%–66%; and 3: >66%.

### Genotyping for candidate SNPs

DNA was extracted from peripheral blood using a standard phenol–chloroform method. A predesigned TaqMan probe (Applied Biosystems, Foster City, CA, USA) was purchased for genotyping of rs4374383 (*MERTK*), rs9380516 (*TULP1*), rs2629751 (*GLT8D2*), rs16851720 (*RNF7*), rs738409 (*PNPLA3*), rs5764455 (*PARVB*), and rs8099917 (*IL28B*). Genotyping was performed according to the manufacturer’s protocol. Valid genotypic data were obtained for rs4374383, rs9380516, rs2629751, rs738409, rs5764455, and rs8099917 in all analyzed subjects. Valid genotypic data could not be obtained for rs16851720, and this SNP was excluded from further analysis. HCV genotypes were determined using by genotype specific PCR as described previously[21].

### Statistical analysis

Categorical data were compared using the χ^2^ and Fisher’s exact tests. Distributions of continuous variables were analyzed using the Student’s *t*-test or the Mann–Whitney *U*-test. A *P* value of <0.05 was considered statistically significant. Factors associated with rapid fibrosis progression were determined using logistic regression analysis. Statistical analyses were performed using the Statistical Package for the Social Sciences software version 18.0 (SPSS Inc., Chicago, IL, USA).

## Results

### Patient characteristics

**Fig 1:**
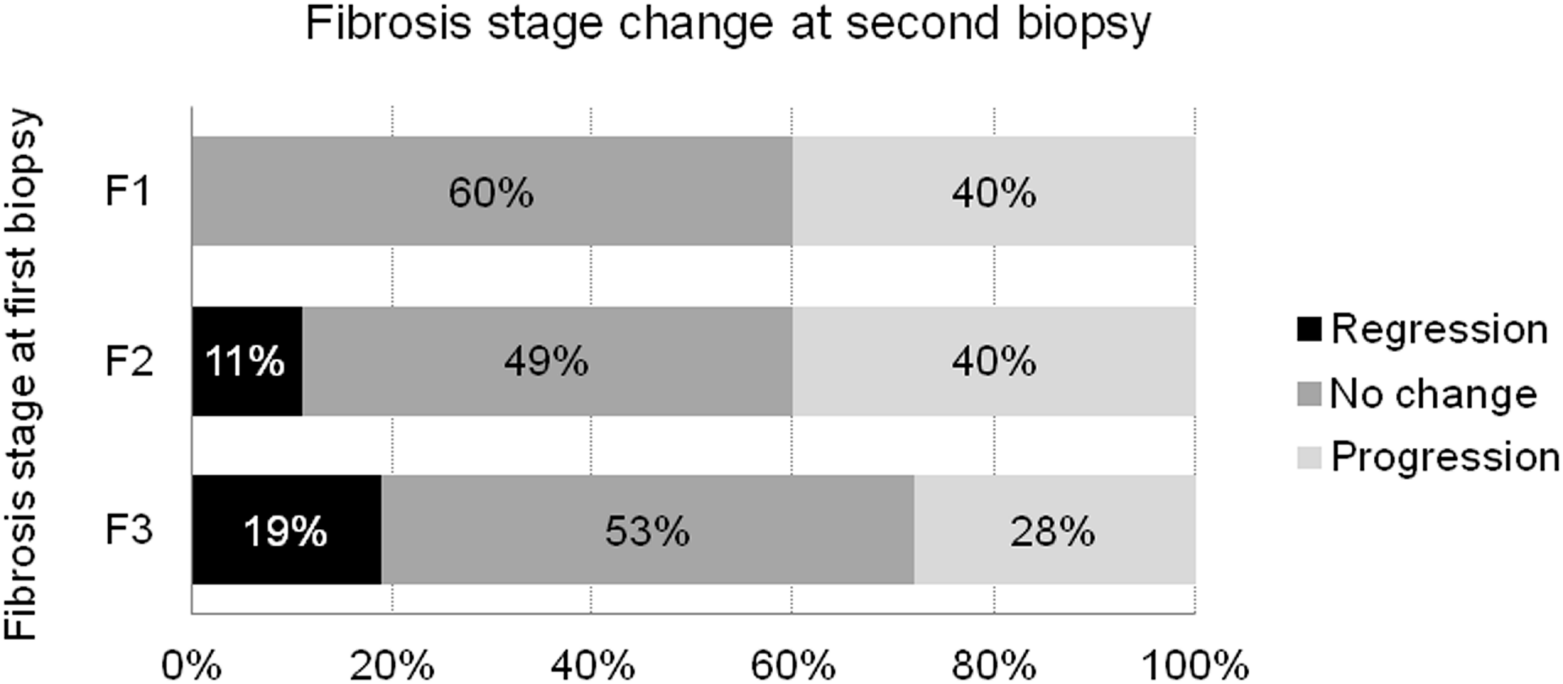
Changes of fibrosis stage over time. Progression of fibrosis was defined as a 1 point or more increase in the METAVIR score. Regression of fibrosis was defined as 1 point or more decrease in the METAVIR score.

Patient characteristics at baseline (the time of first liver biopsy) are shown in Table 1. The mean age was 56.5 years, 40% were male, and 91% were infected with HCV genotype 1. Genotypic frequencies of rs8099917 (*IL28B*), rs738409 (*PNPLA3*), rs4374383 (*MERTK*), rs9380516 (*TULP1*), rs2629751 (*GLT8D2*), and rs5764455 (*PARVB*) are shown in Table 1. The incidence of fibrosis progression at the second biopsy stratified by the stage of fibrosis at baseline is shown in Fig 1. Fibrosis progressed in 38 patients with F1 at baseline (to F2 in 28, to F3 in 8, and to F4 in 2), 21 patients with F2 at baseline (to F3 in 17 and to F4 in 4), and 9 patients with F3 at baseline (Fig 1). Fibrosis progression was observed in 68/176 (40%) patients, and the fibrosis stage did not progress in the remaining 108 patients. Rapid fibrosis progression (FPR ≥ 0.2) was observed in 38 (22%) patients.

**Table 1:** Patients characteristics at baseline.

Patients (n = 176)	
Male/Female	70/106
Age (years)	56.5±9.7
BMI (kg/m^2^)	22.9±3.0
Presence of diabetes mellitus	14(8%)
Albumin (g/ml)	4.2±0.3
AST (IU/l)	63.5±38
ALT (IU/l)	80.6±53
Bilirubin (mg/dl)	0.68±0.3
GGT (IU/l)	62.6±56
Platelets (10^9^/l)	156±50
Cholesterol (mg/dl)	174±34
Triglycerides (mg/dl)	115±64
HCV genotype (1/2)	161/15
Histological findings	
Fibrosis stage (1/2/3)	91/53/32
Steatosis grade (0/1/2/3)	85/78/13/0
Genotype frequencies	
rs8099917:*IL28B* (TT/TG/GG)	109/64/3
rs738409:*PNPLA3* (CC/CG/GG)	52/87/37
rs4374383:*MERTK* (GG/GA/AA)	99/59/18
rs9380516:*TULP1* (CC/CT/TT)	136/37/3
rs2629751:*GLT8D2* (AA/AG/GG)	86/62/28
rs5764455:*PARVB* (GG/AG/AA)	60/87/29
Interval between paired biopsies (years)	6.2±3.8

### Association of SNPs with fibrosis progression rate

The association between SNP genotypes and percentage of patients with fibrosis progression was analyzed. The percentage of patients with fibrosis progression was significantly higher in patients with *IL28B* TG/GG (52%) than in those with *IL28B* TT (29%) (*P* = 0.002). Similarly, it was significantly higher in patients with *PNPLA3* CG/GG compared to CG (44% and 24%, *P* = 0.02) and in patients with *TULP* CT/TT compared to CC (53% and 34%, p = 0.03). No significant association between SNP genotype and fibrosis progression was observed in *MERTK*, *GLT8D2*, and *PARVB*.

**Fig 2:**
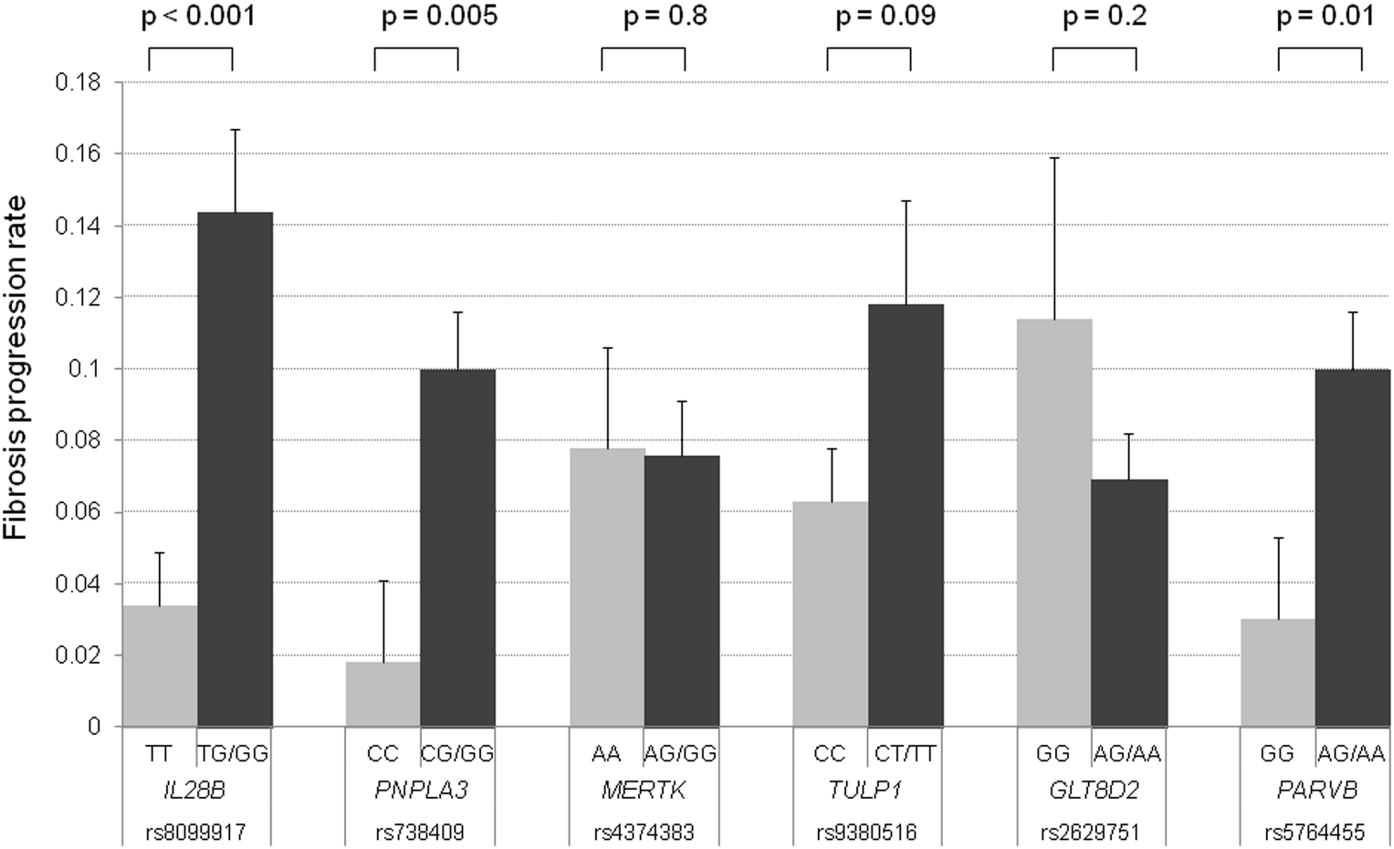
Association of SNPs genotype with fibrosis progression rate. Error bars indicate the standard error.

FPR was calculated by using paired biopsies and was compared with each SNP genotype in all patients (n = 176). The mean FPR was 0.034 in patients with *IL28B* TT, whereas it was 0.144 in those with *IL28B* TG/GG. FPR was significantly higher in patients with *IL28B* TG/GG (*P* < 0.001, Fig 2). Similarly, the mean FPR in patients with *PNPLA3* CC and CG/GG was 0.018 and 0.10, respectively, and the mean FPR in those with *PARVB* GG and AG/AA was 0.03 and 0.10, respectively. FPR was significantly higher in patients with *PNPLA3* CG/GG (*P* = 0.005) and *PARVB* AG/GG (*P* = 0.01); *PARVB* and *PNPLA3* correlated significantly (*P* < 0.001), and the concordance rate of the 2 SNP genotypes was 76%. No significant difference in FPR was observed in patients with *MERTK* AA and AA/AG, *TULP1* CC and CT/TT, and *GLT8D2* GG and AG/AA genotypes. In patients with F1 at first biopsy, FPR was significantly higher in patients with *IL28B* TG/GG than in those with *IL28B* TT (0.161 and 0.044, *P* < 0.001). On the other hand, *PNPLA3* and *PARVB* genotypes were not related to FPR. Similarly, In patients with F2/3 at first biopsy, *PNPLA3* and *PARVB* genotypes were significantly related to FPR, but *IL28B* genotype was not significant.

### Clinical characteristics associated with *IL28B*, *PNPLA3*, and *PARVB* genotypes

We next evaluated the clinical features associated with *IL28B*, *PNPLA3*, and *PARVB* genotypes. The incidence of serum ALT normalization after interferon therapy was significantly higher in patients with *IL28B* TT than in those with *IL28B* TG/GG (*P* = 0.01). On the other hand, the steatosis grade was significantly higher in patients with *PNPLA3* CG/GG and *PARVB* AG/GG than in those with *PNPLA3* CC (*P* < 0.001) and *PARVB* GG (*P* = 0.001), respectively.

### Factors associated with rapid fibrosis progression

Patients with rapid fibrosis progression (n = 38) was compared with remaining patients (n = 138) by univariate analysis. It demonstrated that average ALT after interferon therapy ≥ 40 IU/l, *IL28B* TG/GG, *PNPLA3* CG/GG, and steatosis grade ≥ 1 were risk factors associated with rapid fibrosis progression.

Multivariate analysis demonstrated that *IL28B* TG/GG [hazard ratio (HR): 3.9, 95% confidence interval (CI): 1.7–8.9, *P* = 0.001], *PNPLA3* CG/GG (HR: 3.1, 95% CI: 1.1–9.5, *P* = 0.02), and average ALT after interferon therapy ≥ 40 IU/l (HR: 4.2, 95% CI: 1.7–10, *P* = 0.002) were independent factors associated with rapid fibrosis progression (Table 2).

**Table 2:** Factors associated with rapid fibrosis progression.

		Univariate			Multivariate		
		Hazard ratio	95% CI	p Value	Hazard ratio	95% CI	p Value
Sex	Female	1					
	Male	0.9	0.5–2.0	0.9			
Age (by every 10 years)		0.9	0.7–1.3	0.7			
BMI (kg/m2)		1.0	0.9–1.2	0.5			
Presence of diabetes mellitus		2.2	0.7–6.9	0.2			
Albumin (g/ml)		1.0	0.3–3.4	0.9			
AST (by every 40 IU/l)		0.8	0.5–1.2	0.3			
ALT (by every 40 IU/l)		0.9	0.7–1.2	0.5			
Bilirubin (mg/dl)		0.7	0.2–2.5	0.7			
GGT (by every 10 IU/l)		1.0	1.0–1.1	0.3			
Platelets (109/l)		1.0	0.9–1.1	0.7			
Cholesterol (by every 10 mg/dl)		1.0	0.9–1.1	0.3			
Triglycerides (by every 10 mg/dl)		1.0	1.0–1.1	0.1			
ALT after interferon therapy (IU/l)	< 40 IU/l	1			1		
	≥ 40 IU/l	5.3	2.2–12.8	<0.001	4.2	1.7–10	0.002
HCV genotype	1	1					
	2	0.5	0.1–2.5	0.4			
Histological findings							
Fibrosis stage	F1	1					
	F2	0.9	0.4–1.9	0.7			
	F3	0.5	0.2–1.5	0.2			
Steatosis grade	0	1			1		
	1–3	2.4	1.1–5.2	0.02	1.7	0.7–4.0	0.2
SNP Genotype							
rs8099917: *IL28B*	TT	1			1		
	TG/GG	3.8	1.8–8.0	<0.001	3.9	1.7–8.9	0.001
rs738409: *PNPLA3*	CC	1			1		
	CG/GG	3.4	1.3–9.3	0.02	3.1	1.1–9.5	0.04
rs4374383: *MERTK*	AA	1					
	AG/GG	1.4	0.4–5.2	0.6			
rs9380516: *TULP1*	CC	1					
	CT/TT	2.1	0.9–4.7	0.06			
rs2629751: *GLT8D2*	GG	1					
	AG/AA	0.8	0.3–2.0	0.6			
rs5764455: *PARVB*	GG	1					
	AG/AA	2.3	0.9–5.3	0.06			

### Evaluation of fibrosis progression risk by combining factors associated with rapid fibrosis progression

**Fig 3:**
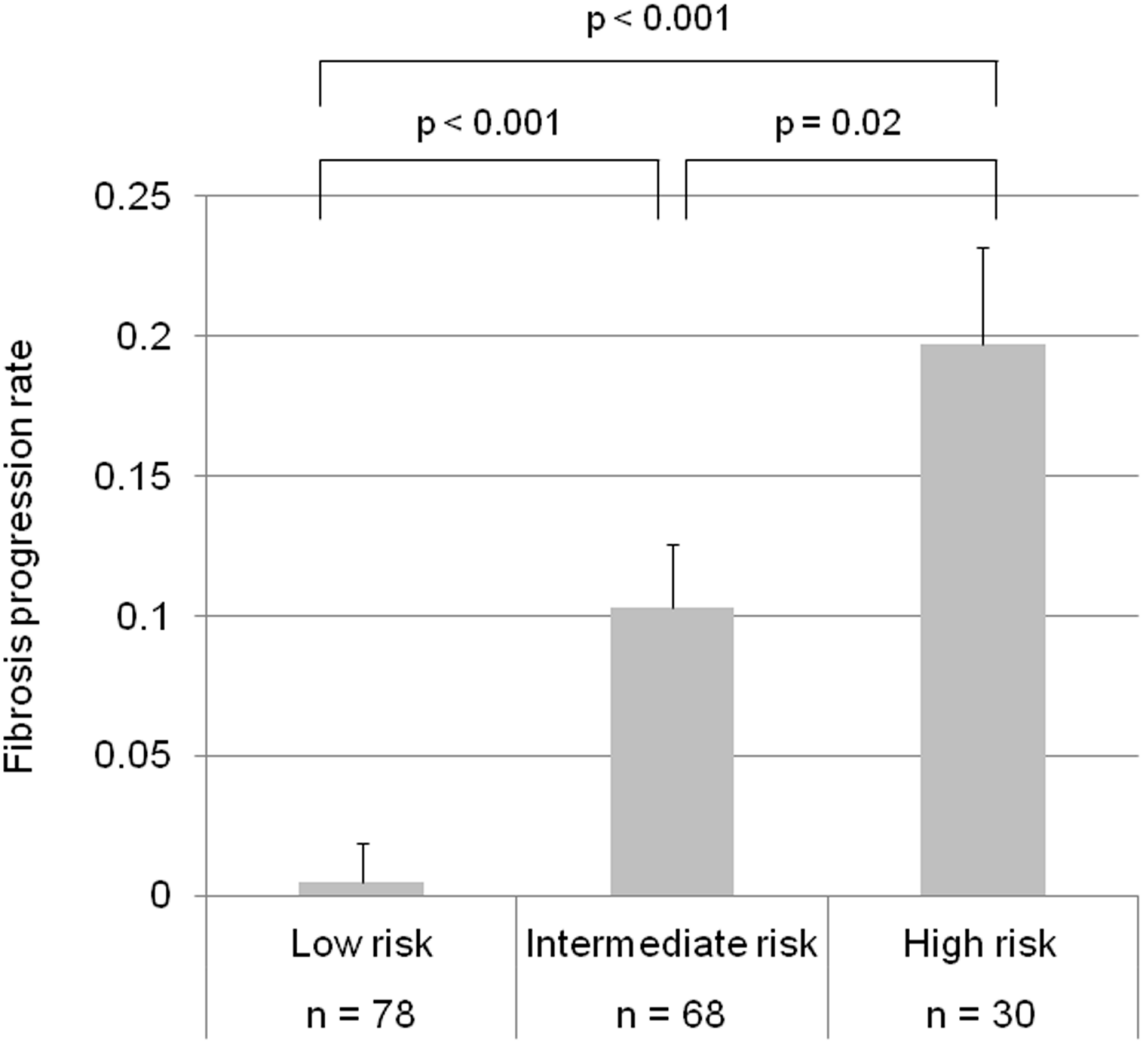
Association between fibrosis progression rate and combining risk analysis (FPR score). *IL28B* TG/GG, *PNPLA3* CG/GG, and average ALT after interferon therapy ≥ 40 IU/l were scored as 1 point. Similarly, *IL28B* TT, *PNPLA3* CC, and average ALT after interferon therapy < 40 IU/l were scored as 0 point. Patients scored 0–1, 2 and 3 were defined as a low, intermediate, and high risk group, respectively. Error bars indicate the standard error.

*IL28B* and *PNPLA3* genotypes, and average ALT levels after interferon therapy were independent factors associated with rapid fibrosis progression, hence we developed a scoring system (FPR-score) for prediction of fibrosis progression based on the multivariate analysis. *IL28B* TG/GG, *PNPLA3* CG/GG, and average ALT after interferon therapy ≥ 40 IU/l were scored as 1 point. Similarly, *IL28B* TT, *PNPLA3* CC, and average ALT after interferon therapy < 40 IU/l were scored as 0 point, and these three factors were combined. The FPR score identified a subgroup of patients with a high (score of 3), intermediate (score of 2), or low (score of 0–1) risk of fibrosis progression. The mean FPR was 0.005 in patients with a low risk, whereas it was 0.103 and 0.197 in those with an intermediate and high risk, respectively. The FPR was significantly higher in patients with an intermediate (*P* < 0.001) and high risk (*P* < 0.001) than in those with a low risk (Fig 3). The FPR was also significantly higher in patients with a high risk compared to those with an intermediate risk (*P* = 0.02). Patients with rapid fibrosis progression were 3 (2/78), 28 (19/68), and 50% (15/30) in a low, intermediate, and high risk group, respectively. When analyzed by the incidence of rapid fibrosis progression, it still differed significantly between three groups (*P* < 0.001).

## Discussion

Through analysis of long-term data, we demonstrated that genotypes of *IL28B* and *PNPLA3* are independent predictors of rapid fibrosis progression in patients with chronic hepatitis C who failed to achieve SVR following interferon-based therapy. A major strength of the present study was the assessment of longitudinal progression of liver fibrosis within each individual by paired biopsies. Thus we had the unique opportunity to establish the association of *IL28B* and *PNPLA3* genotypes and rapid fibrosis progression. Moreover, we developed a fibrosis progression (FPR)-score by combining *IL28B* and *PNPLA3* genotypes and ALT values, which stratified patients into a low, intermediate and high-risk group for fibrosis progression. FPR score may become, if validated in other external patient cohorts, an easy-to-use clinical tool to guide decision-making in daily clinical practice and to tailor indication for (expensive) antiviral therapy with new DAA’s.

Several other genes, which may be associated with fibrosis progression, have been proposed in previous studies. European GWAS have identified SNPs in *MERTK*, *GLT8D2*, *TULP1*, and *RNF7* as the genetic factors responsible for liver fibrosis progression in chronic hepatitis C during the natural course[12]. However, the association between these SNPs and fibrosis progression was not observed in our study. On the other hand, *IL28B* TG/GG was significantly associated with liver fibrosis progression. Although several studies analyzed the association between fibrosis progression and the *IL28B* genotype[9,10,11], most were cross-sectional studies analyzing liver biopsies at single time points[9,10]. Noureddin et al. analyzed paired biopsies from an NIH cohort[22] and the HALT-C cohort[23] and reported that the *IL28B* genotype is not associated with fibrosis progression[11]. The most important difference from our study was that patients of the NIH cohort did not receive interferon therapy and that approximately 40% of the HALT-C cohort presented with cirrhosis. In our study, we focused on non-SVR patients following interferon therapy, using paired biopsies, and excluded patients with cirrhosis at baseline because they were not suitable for the assessment of fibrosis progression. These differences in patients clinical backgrounds including differences in race, HCV genotype as well as study designs may likely explain the conflicting results.

The significance of *IL28B* genotypes on fibrosis progression was further supported by the association of *IL28B* TT and normalization of ALT levels in non-SVR patients. Several studies revealed that a high serum ALT level is a risk factor for rapid fibrosis progression[11,22,24,25], and normalization of the ALT level after interferon therapy is associated with histological improvement and hepatocellular carcinoma development even in non-SVR patients[26,27,28]. Our results suggest that the lower risk of fibrosis progression observed in *IL28B* TT patients partly results from the suppressive effect of interferon on ALT levels in these non-SVR patients.

Another important finding of the present study is that the *PNPLA3* G allele was associated with time-dependent progression of fibrosis and steatosis grade. Hepatic steatosis is a common pathological finding in patients with chronic hepatitis C[29]. Many studies have analyzed the association between steatosis and liver fibrosis, and most of them, including a large-scale meta-analysis, have shown a positive correlation [9,11,30,31,32,33]. Since *PNPLA3* has been recognized as a genetic determinant of liver fat content in patients with both NAFLD[13,14,15,16] and alcoholic liver disease[34,35], it was hypothesized that this SNP is also associated with steatosis and fibrosis progression in patients with chronic hepatitis C. Although previous studies reported a positive correlation between the *PNPLA3* genotype and steatosis or fibrosis progression in patients with chronic hepatitis C[19,20], this correlation was controversial due to their cross-sectional study design. Our results obtained by analysis of paired biopsy specimens provide robust evidence to support the role of the *PNPLA3* genotype in fibrosis progression and warrant further studies to explore the mechanistic role of *PNPLA3* in fibrosis progression in patients with chronic hepatitis C.

In the present study, it was reported that *PNPLA3* genotype was associated with fibrosis progression only in diabetic patients[36]. In our study where vast majority of patients did not have diabetes, *PNPLA3* genotype was a significant factor of fibrosis progression. Since the number of diabetic patients was too small to perform a sub-analysis, a future study is needed to evaluate the different impact of *PNPLA3* genotype in diabetic and non-diabetic patients.

There are some limitations in the present study. Although the changes in fibrosis stage overtime were analyzed by long-term observation period, 60% of patients did not have fibrosis progression. Since the stage of fibrosis was graded by 4 levels pathologically (F1 to F4), a marginal increase or decrease of fibrosis fall into the same stage, which may have lead to a high rate of patients without fibrosis progression. Another limitation is that patients with F3 might leave limited space for disease progression as compared with F1-2 which might have affected the impact of genotypes. A future study is needed to evaluate the different impact of genotypes in patients with and without advanced fibrosis at the start of observation. Few patients had fibrosis regression though interferon therapy was failed. Interferon might have played a role but the precise mechanism underlining fibrosis regression during persistent HCV infection is not clear. A previous report has indicated that fibrosis could regress in some proportion of untreated and non-SVR patients[37]. Host factors may be related to the natural or interferon-induced fibrosis regression. This may be another target of future investigation.

In conclusion, *IL28B* and *PNPLA3* genotypes are associated with rapid fibrosis progression in patients with chronic hepatitis C. The FPR score identifies HCV patients who has a high risk of fibrosis progression and require urgent antiviral treatment.
